# Supplementary material for: Localized Wnt-signaling promotes asymmetric NuMA-dependent oriented divisions and unequal apportioning of mitochondria
Source: Nat Commun. 2025 Nov 27;16:10690. doi: 10.1038/s41467-025-65775-z (PMC12660846; doi:10.1038/s41467-025-65775-z)
Supplement: Supplementary file 1 — Supplementary Information [file 41467_2025_65775_MOESM1_ESM.pdf]

Localized Wnt-signaling promotes asymmetric NuMA-dependent oriented divisions and unequal apportioning of mitochondria

Eli et al.

SUPPLEMENTARY INFORMATION

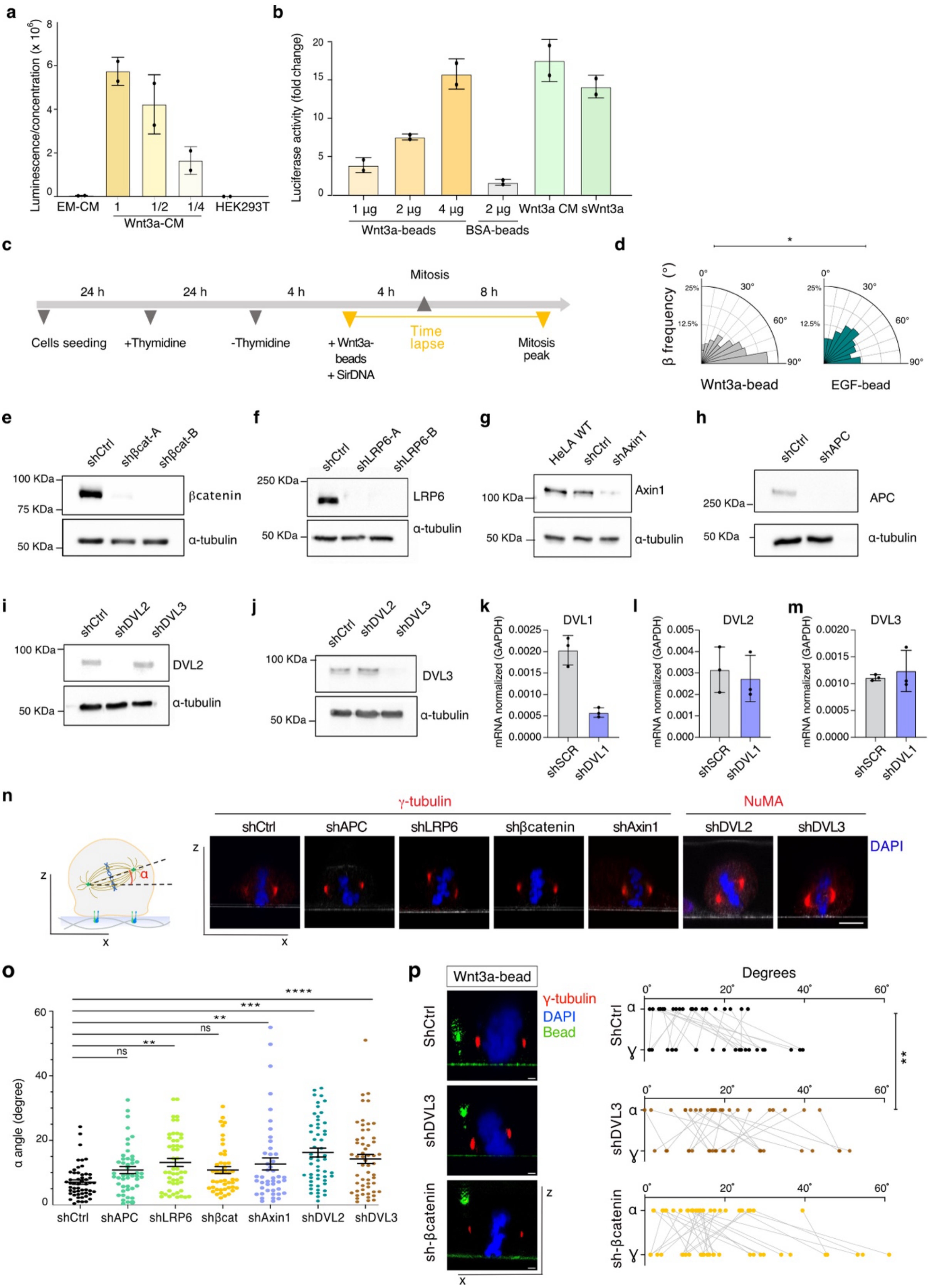

Supplementary Figure 1. Characterization of HeLa cell lines silenced for canonical Wnt effectors.

(a) Quantification of TOP-FLASH assays conducted in HEK293T-7xTCF-Luciferase cells treated with

different concentration of Wnt3a-conditioned medium (Wnt3a-CM). Luminescence was normalized on lysate concentration. Cells with empty-conditioned media (EM-CM) or parental HEK293T cells (293T) were used as negative controls. Means ± SD are shown. **(b)** Histogram of TOP-FLASH assay luminescence fold change compared to EM-CM of HEK-293T-7xTCF-Luciferase cells seeded with different amounts of Wnt3a-coated beads or BSA-coated beads. Wnt3a-conditione media (CM) or 200 μg/μl purified Wnt3a ligands (soluble Wnt3a, sWnt3a) were used as positive controls. Means ± SD are shown. **(c)** Schematic representation of the protocol used for time-lapse videorecording of HeLa cells dividing in contact with Wnt3a-coated beads. HeLa cells were synchronized by single thymidine block and, after release, Wnt3a beads and SirDNA were added to the cells 4 hours before the expected mitotic peak. **(d)** Rose plots of the β angle distribution measured in time-lapse experiments with EGF-coated bead of HeLa cells. Wnt3a-beads were used as a positive control. Unpaired T-test was performed for three independent experiments with n = 129 for Wnt3a-beads and n = 135 for EGF-beads. *, p-value< 0.05 (p-value =0.0271). (**e-f)** Immunoblot of lysates of HeLa cells stably depleted of β-catenin, LRP6 by lentiviral transduction showing the efficiency of two shRNA-based knock-down. (**g-h)** Immunoblot of lysates of HeLa cells stably depleted of Axin1, APC by lentiviral transduction. **(i-j)** Immunoblot of lysates of HeLa cells stably depleted of DVL2 and DVL3 by shRNA interference. In the two cell lines, the specificity of isoform downregulation was assessed by testing the expression of both Dvl2 and Dvl3. For all immunoblots, α-tubulin was used as loading control. **(k-m)** Gene expression of DVL1, DVL2 and DVL3 evaluated by RT-qPCR in HeLa cell lines depleted of DVL1 by shRNA interference. For each DVL gene, mRNA levels were normalized to the value of mRNA levels measured in shCtrl HeLa cells. Means ± SD are shown from three independent experiments. **(n)** Left: Cartoon depicting a metaphase HeLa cell in which the α-angle (in red) is measured in the X-Z axis as a line passing through the spindle poles (green) and the substratum. Right: Representative confocal x-z sections of the HeLa cells with indicated knock-downs used for the quantification in (o). Cells were stained with γ-tubulin or NuMA (red) to visualize spindle poles, and DAPI (blue) to image the metaphase plate. **(o)** Dot-plot with spindle angle distributions of mitotic HeLa cells depleted of APC, LRP6, β-catenin, Axin1, DVL2 or DVL3. Means ± SEM are shown for 3 independent experiments with shCtrl n=53, shAPC n=47,

shLRP6 n=50, shβ-catenin n=46, shAxin1 n=45, shDVL2 n=55 and shDVL3 n=57. **p-value < 0.01, ***p-value< 0.001, ****p-value< 0.0001 by the Ordinary one-way ANOVA test (shAPC p-value= 0.193, shLRP6 p-value= 0.0052, shβ-catenin p-value= 0.198, shAxin1 p-value=0.0156, shDvl3 p-value=0.0001). (**p**) Left: Confocal x-z sections of HeLa cells with the indicated depletion dividing in contact with Wnt3a-beads. Cells were stained with γ- tubulin (red) to visualize the spindle poles and DAPI (blue) for the DNA. Beads are visible by autofluorescence (green), coverslips are visible in green. Scale bars, 2 μm. Right: Quantification of the relationship between the α and γ angles in metaphase HeLa cells dividing in contact with Wnt3a-beads shown on the left (see Fig. 1e for angle definition). For each condition, line-plots with the distributions of α and γ angles from 3 independent experiments are shown with n= 23 for shCtrl; n= 23 for shDVL3; and n= 31 for sh-βcatenin. Only the significant difference by Mann-Whitney non-parametric t-test is shown, **, p-value= 0.0044.

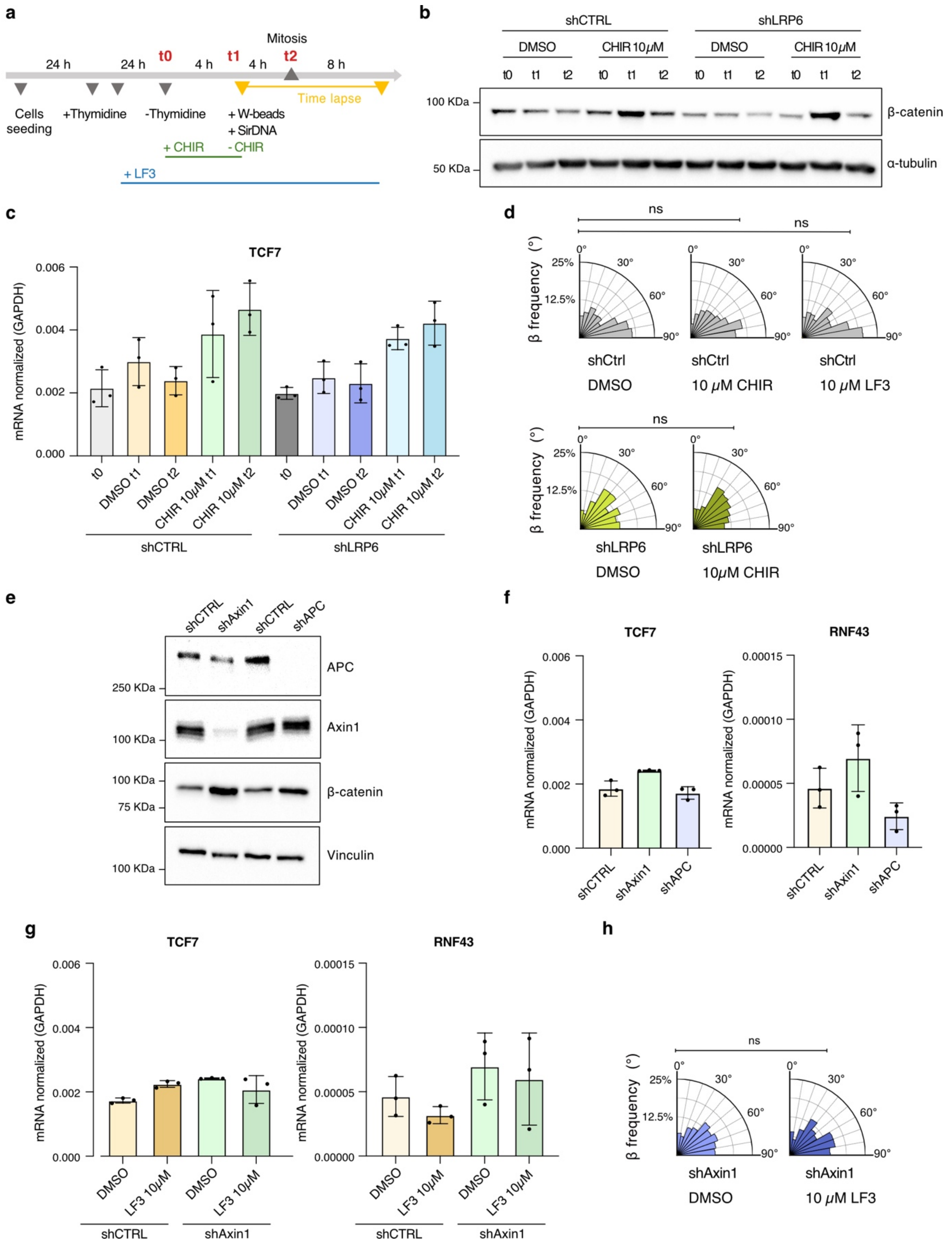

Supplementary Figure 2. Impact of Wnt transcription activation on spindle orientation towards Wnt3a-beads. (a) Schematic representation of the protocol used for time-lapse videorecording of HeLa cells dividing in contact with Wnt3a-coated beads coupled to Wnt transcriptional modulation. To activate Wnt gene transcription, HeLa cells were synchronized by single thymidine block and, after

release, treated with 10 μM CHIR-99021 (time point t0) for 4 hours. After CHIR-99021 wash-out, Wnt3a beads and SirDNA were added to the cells (time point t1) 4 hours before the expected mitotic peak (time point t2). To assess the effect of Wnt gene transcription inactivation, after about 4 hours of thymidine addition (about 24 hours before the mitotic peak), 10 μM LF3 were added to the cultured HeLa cells. Thymidine was washed-out about 16 hours later (timepoint t0) and Wnt3a-beads seeded at the mitotic peak (timepoint t2). Timelapse videorecording was protracted for about 12 hours from t1. (**b**) Immunoblot analysis of expression levels of β-catenin at t0, t1 and t2 in shCtrl and shLRP6 HeLa cells treated with 10 μM CHIR-99021 or DMSO as control. (**c**) Gene expression of the Wnt target gene TCF7 evaluated by RT-qPCR in shCtrl and shLRP6 HeLa cells treated with 10 μM CHIR-99021 or DMSO as control at the time points t0, t1 and t2. For each condition n=3 biological replicates were performed. Means ± SD is shown. (**d**) Top: Rose-plots of the β angle distribution of shCtrl HeLa cells treated with DMSO or for 4 hours with 10 μM CHIR-99021 or for 24 hours with 10 μM of the Wnt-transcription inhibitor LF3, in three independent experiments, with n = 700 for DMSO; n = 485 for CHIR-99021 and n = 325 for LF3. By applying the Kruskal–Wallis non-parametric test, no statistical significance was scored between conditions (p = 0.6384). Bottom: Rose-plots of the β angle distribution of shLRP6 HeLa cells treated for 4 hours with DMSO or with 10 μM CHIR-99021. Data are shown from 3 independent experiments, with n = 218 for DMSO and n = 306 for 10 μM CHIR-99021. By applying the Mann-Whitney nonparametric t-test, no statistically relevant difference was scored among conditions (p = 0.6141). (**e**) Immunoblot analysis of the expression levels of APC, Axin1 and β-catenin in shCtrl, shAxin1 and shAPC HeLa cell lines. Vinculin was used as a loading control. (**f**) Gene expression of TCF7 and RNF43 evaluated by RT-qPCR in shCtrl, shAxin1 and shAPC HeLa cell lines. For each condition n=3 biological replicates were performed. Means ± SD is shown. (**g**) Gene expression of TCF7 and RNF43 evaluated by RT-qPCR in shCtrl and shAxin1 HeLa cells treated for 24 hours with 10 μM LF3. For each condition n=3 biological replicates were performed. Means ± SD is shown. (**h**) Rose-plots of the β angle distribution of shAxin1 HeLa cells treated for 24 hours with 10 μM LF3 or DMSO as control evaluated in three independent experiments, with n = 412 for DMSO and

n= 365 for LF3. By applying Mann-Whitney non-parametric t-test, no statistically relevant difference was scored among conditions (p = 0.6371).

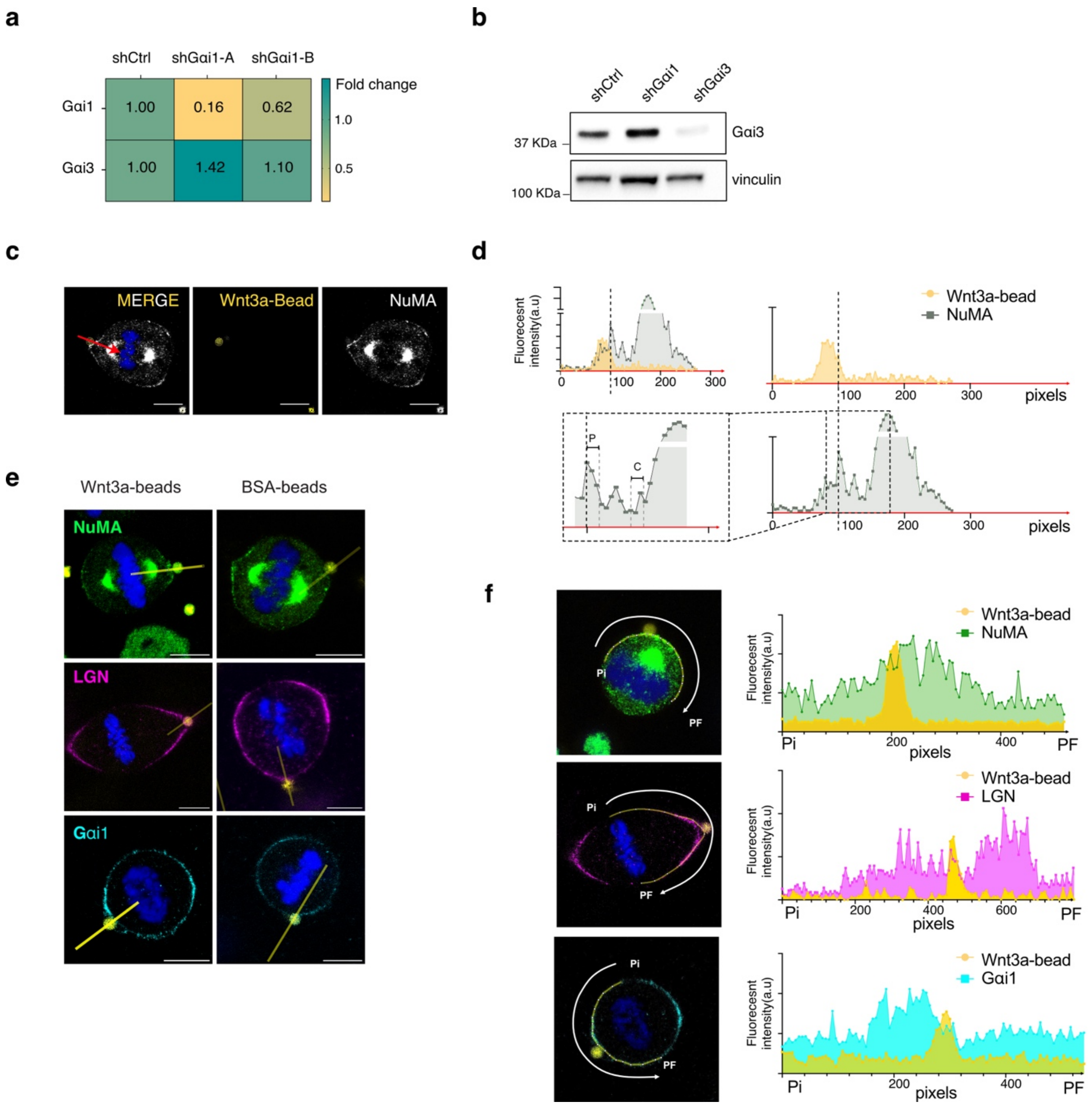

Supplementary Figure 3. (a) Gene expression of Gαi1 and Gαi3 evaluated by RT-qPCR in HeLa cell lines silenced for Gαi1 and Gαi3, respectively. For each Gαi isoform, mRNA levels were normalized to the value of mRNA levels measured in shCtrl HeLa cells. **(b)** Gαi3 immunoblot of HeLa cells stably depleted of Gαi1 and shGαi3. Vinculin was used as loading control. **(c)** Confocal images of mitotic HeLa cells dividing in contact with Wnt3a-coated beads (yellow) stained for NuMA (white). A line-scan was drawn at the cortical Wnt3a-bead contact site (red arrow). **(d)** Histogram of NuMA signal

intensity (grey) and bead autofluorescence (yellow) through the line scan. The level of cortical NuMA accumulating at the Wnt3a-bead was calculated based on the bead position, as the ratio between the signal intensity at the bead contact side (P) and the signal intensity in the cytosol (C). **(e)** Representative confocal images of metaphase HeLa cells in contact with Wnt3a-beads (left panels) or BSA-bead (right panels). Cells are stained for NuMA (green, top panels), LGN (magenta, middle panels) and Gαi1 (cyan, lower panels). Scale bar, 10 µm. (**f**) Left: confocal images of mitotic HeLa cells dividing in contact with Wnt3a-coated beads (yellow) stained for NuMA (green), LGN (magenta) and Gαi1 (cyan). The cortical segments used for profile measurements are marked in yellow, and the direction of profile plotting is indicated with a white arc from the initial position (Pi) to the final one (PF). Right: cortical fluorescence intensity profile of NuMA, LGN and Gαi1 (based on color code on the left) and bead autofluorescence (yellow) along the mitotic cortex form Pi to PF. To evaluate protein distribution profiles, a segmented line 6 µm in depth was traced from Pi to PF.

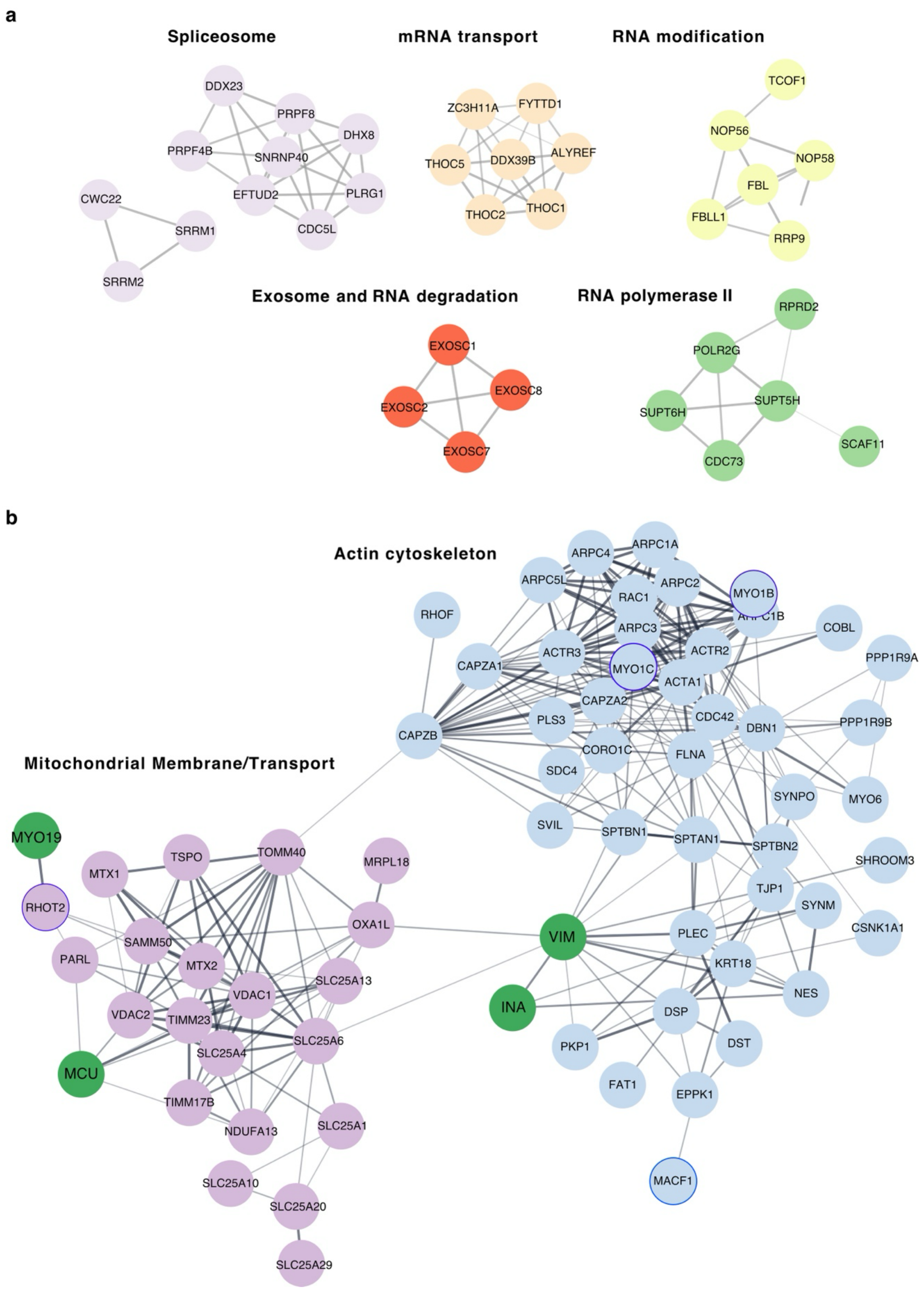

Supplementary Figure 4. (a) Network analysis of RNA-related ontologies derived from the Wnt3a-bead proteomic. Protein clustering identifies different classes of RNA-binding proteins enriched at Wnt3a-coated beads. Nodes indicate proteins, edges represent annotated evidence of physical

interactions among them. The MCL (Markov Cluster Algorithm) clustering was performed with a granularity parameter of 15. To identify classes of proteins that are over-represented in a large set of proteins, a functional enrichment is performed. **(b)** Protein-protein interaction network of mitochondrial and actin-related GO terms from the Wnt3a-bead proteomic. Nodes indicate proteins, edges represent evidence of physical interactions among them. Proteins belonging to actin cyctoskeleton are colored in lightblue, proteins belonging to mitochondrial terms are colored in violet. Shared proteins among the two categories are colored in green.

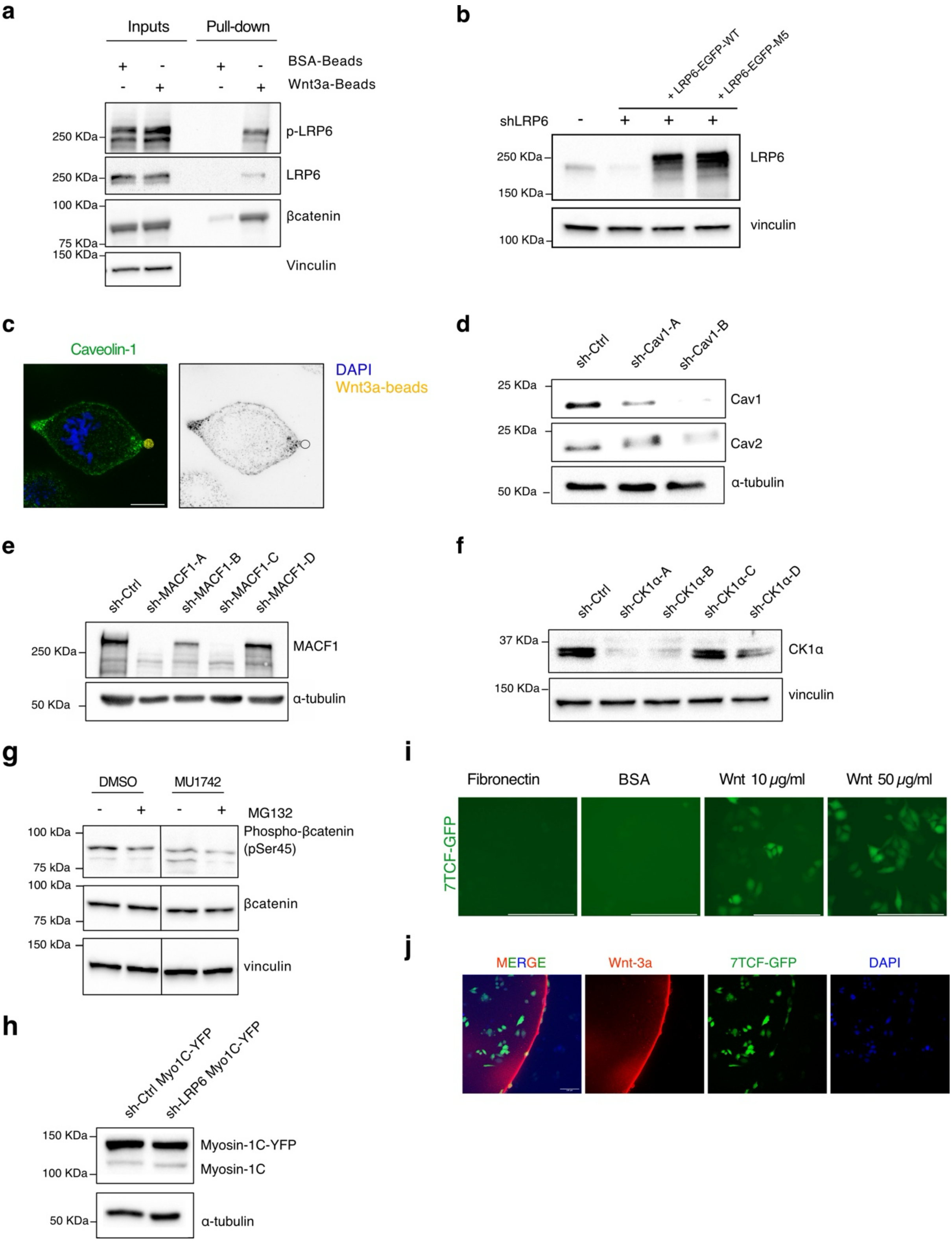

Supplementary Figure 5. (a) Pull-down assay with Wnt3a-beads or BSA-beads conducted with HeLa cells synchronized in metaphase. After cell lysis, species retained on beads were separated on SDS-PAGE and immunoblotted with the indicated antibodies. 20 µg of mitotic lysate were loaded as input, vinculin was used as loading control. **(b)** Immunoblot of cellular lysates of HeLa cells stably depleted

of LRP6 and transfected with the sh-resistant LRP6-EGFP rescue constructs indicated, i.e. LRP6 wild-type and LRP6-M5, carrying 5 mutations on the CK1/GSK3β phosphorylation sites. Vinculin was used as a loading control. (**c**) Confocal images of metaphase HeLa cells in contact with a Wnt3a-coated bead (yellow) stained with caveolin-1 (green). Scale bar, 10 μm. **(d)** Immunoblot analysis of expression levels of caveolin-1 and caveolin-2 in HeLa cells stably depleted of caveolin-1 (with two different shRNA sequences, A and B). Reduction of caveolin-1 levels suffices to downregulate caveolin-2. HeLa cells expressing shCav1-B were used for the experiments of Fig. 5b. α-tubulin was used as loading control. **(e)** Immunoblot analysis of HeLa cells stably depleted of MACF1 with four different shRNA sequences. HeLa cells expressing shMACF1-A were used for the experiments of Fig. 5B. α-tubulin was used as loading control. **(f)** Immunoblot analysis of HeLa cells stably depleted of Ck1α with four different shRNA sequences. HeLa cells expressing shCK1α-A were used for the experiments of Fig. 5c. Vinculin was used as loading control. **(g)** Immunoblot of β-catenin and pS45-β-catenin in lysates of HeLa cells treated with DMSO or 10 μM of the CK1 inhibitor MU1742 for three hours, with and without MG132. Vinculin was used as loading control. **(h)** Immunoblot analysis of myosin1C expression in HeLa cells shCtrl and shLRP6 used for the TIRF experiment, transfect with myosin1C-YFP. α-tubulin was used as loading control. **(i)** GFP widefield fluorescent images of HeLa cells expressing 7TCF-GFP reporter seed on platform coated with fibronectin, BSA or purified Wnt3a at different concentration. Scale bar, 200 μm. **(j)** Confocal images of 7xTCF-GFP HeLa cells seeded on Wnt3a platforms. Wnt3a is diluted in an infrared-BSA shown in red and dropped on the surface. Its absorption is visualized by the 7TCF-GFP-reporter of Wnt activation. Scale bar, 100 μm.

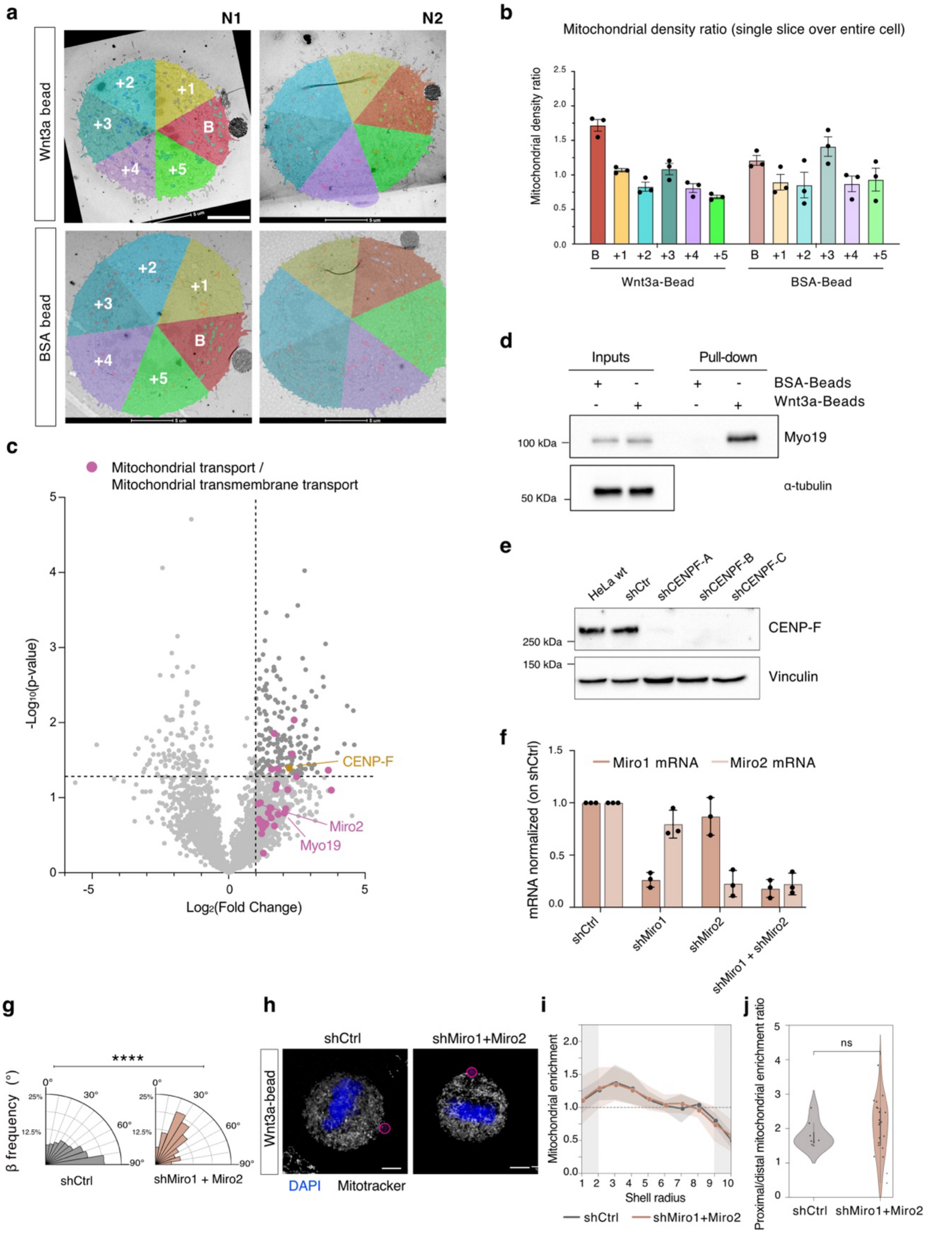

Supplementary Figure 6. Mitotic enrichment of mitochondria at Wnt3a-bead contact site in HeLa cells. (a) Representative transmission electron microscopy images of HeLa cells dividing in contact with Wnt3a-coated beads (upper panel) or BSA-coated beads (lower panel), with two representative examples for each condition (N1, N2). Each cell was divided into six circular sectors of 60° and color-

coded accordingly. *B* marks the sector proximal to the bead. Scale bar, 5 μm. **(b)** Histogram showing the mitochondrial density ratio in each sector relative to the overall mitochondrial density of the entire cell. The ratio was calculated as the mitochondrial density in each circular sector divided by the total cell mitochondrial density, starting from the sector proximal to the bead (shaded in red, labeled *B*) and proceeding in an counter-clockwise direction (+1, +2, +3, +4, +5). Data represent mean ± SD for three metaphase HeLa cells in contact with Wnt3a-coated beads (darker bars) or BSA-coated beads (lighter bars). **(c)** Pull-down mass spectrometry data (Wnt3a-beads vs BSA-beads) with proteins belonging to GO biological processes of *Mitochondrial transport* and *Mitochondrial transmembrane transport* are coloured in magenta. The position of Myosin19 and Miro2 are highlighted, together with CENP-F (green). **(d)** Magnetic pull-down assays with Wnt3a-coated or BSA-coated beads conducted in metaphase HeLa cell extracts. Species retained on beads were separated on SDS-PAGE and immunoblotted with Myosin19 antibody. α-tubulin was used as a loading control. (**e**) Immunoblot of lysates of HeLa cells stably depleted of CENP-F by lentiviral transduction showing the efficiency of three shRNA-based knock-down. Vinculin was used as loading control. (**f**) Histograms of gene expression levels of Miro1 (RHOT1 gene) and Miro2 (RHOT2 gene) evaluated by RT-qPCR in HeLa cell lines depleted of Miro1, Miro2 or both by shRNA interference. For each gene, mRNA levels were normalized to the value of mRNA levels measured in shCtrl HeLa cells. Means ± SD are shown from three independent experiments. (**g**) Rose-plots of β-angle distribution shown for two independent experiments, with shCtrl n = 212, shMiro1+shMiro2 n = 256. The Kruskal–Wallis non parametric t-test was applied. ****; p=value < 0.0001. (**h**) Representative 3D reconstruction of mitochondrial networks in metaphase HeLa cells doubly depleted of Miro1 and Miro2 dividing in contact with Wnt3a-beads. ShCtrl expressing cells were used as control. Cells were stained with DAPI (blue) to visualize DNA, and Mitotracker CMXRos Red M7512 (white) to visualize mitochondria. Beads are visible by autofluorescence (magenta circle). Scale bar, 5 μm. (**i**) Normalized mean fluorescence intensity (MFI) profiles of the mitochondrial signal along the shells for shCtrl (gray) and shMiro1 + shMiro2 (salmon) HeLa cells dividing in contact with Wnt3a-beads. Data are shown as Mean ± Standard Deviation of n = 8 for shCtrl and n = 21 for shMiro1+shMiro2, from 3 independent experiments. (**j**) Single dots indicate

values of individual cells; black lines indicate median and interquartile range. shCtrl: Minima = 1.497921485064299; maxima = 2.604132428383956; centre = 1.6441888333264498; Q1 = 1.5882588912296738; Q3 = 1.8822479735580113; Q1 percentile = 25; Q3 percentile = 75. shMiro1-2: Minima = 0.4153578107645802; maxima = 3.839505039371658; centre = 2.163568667300858; Q1 = 1.512682765601133; Q3 = 2.591989807745979; Q1 percentile = 25; Q3 percentile = 75. p = 0.429, not significant by the Mann-Whitney non-parametric t-test.

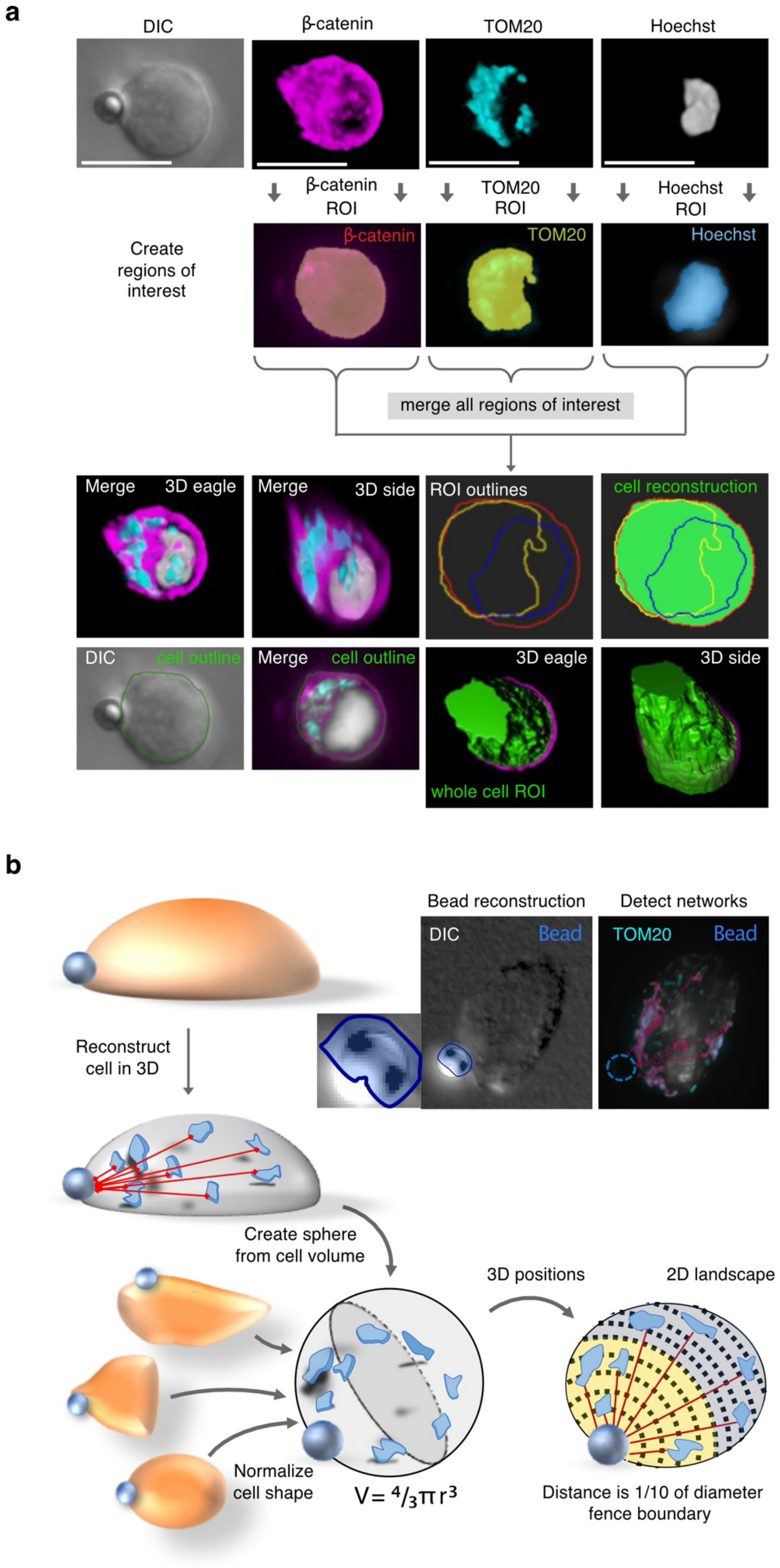
a
DIC
β-catenin
TOM20
Hoechst
β-catenin ROI
TOM20 ROI
Hoechst ROI
Create regions of interest
β-catenin
TOM20
Hoechst
merge all regions of interest
Merge
3D eagle
Merge
3D side
ROI outlines
cell reconstruction
DIC
cell outline
Merge
cell outline
3D eagle
whole cell ROI
3D side
b
Bead reconstruction
Detect networks
DIC
Bead
TOM20
Bead
Reconstruct cell in 3D
Create sphere from cell volume
3D positions
2D landscape
Normalize cell shape
V= 4/3π r3
Distance is 1/10 of diameter
fence boundary

Supplementary Figure 7. Schematic pipeline for defining the 3D cellular reconstruction region of interest. ***(a)*** Representative image panel showing DIC (grey), β-catenin (purple), Tom20 (turquoise), and Hoechst (white) staining. The first row presents images in extended focus, while the third and fourth? rows display 3D views from the top and side, respectively. Scale bar: 10 µm. Second row: Regions of interest (ROI) for each staining were produced in an intensity-dependent manner. Third row: These regions of interest were combined and merged to create a comprehensive region of interest encompassing the entire cell. (**b**) Cell and bead structures were reconstructed in 3D, with mitochondrial networks detected and their distances to the bead measured. The cell volume was converted to a sphere to normalize the distance to the cell size, creating fences at intervals of one-tenth of the spherical diameter. This method allowed for the mapping of the 3D positions of mitochondrial networks onto a 2D landscape.

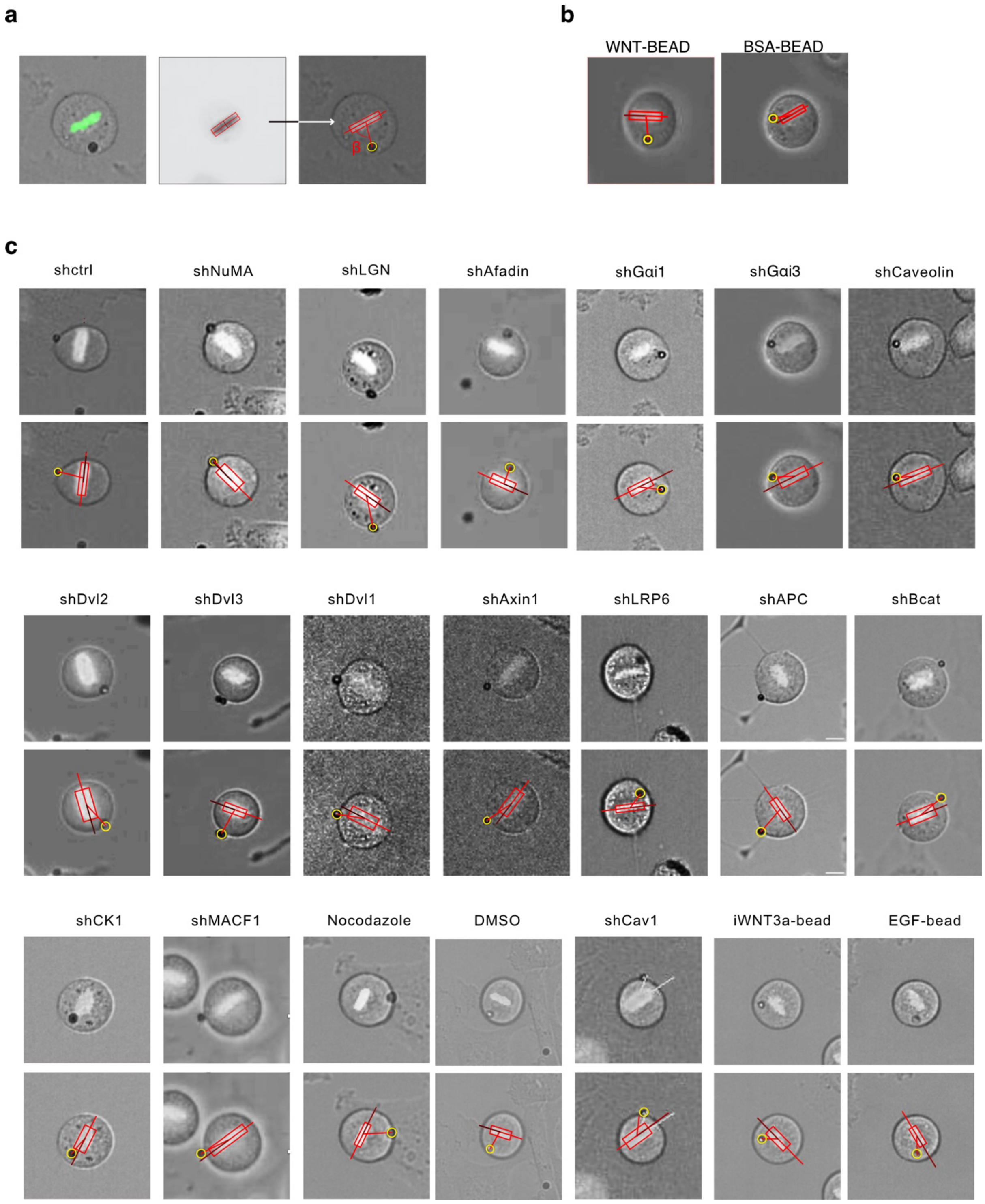

Supplementary Figure 8. Representative images of metaphase HeLa cells in contact with Wnt3a-beads used for division orientation analyses. (**a**) Scheme of the workflow employed to measure the β-angle formed by a line passing through the beads and the metaphase plate. The metaphase plate orientation (boxed in red) was visualized by the fluorescence signal of labelled DNA (green) of the last metaphase frame from videorecording of HeLa cells dividing in contact with functionalized beads

(circled in yellow). (**b**) Representative images of β-angle measurements of HeLa cells dividing in contact with Wnta3a-beads or BSA-beads. (**c**) Representative images and β-angle determination of HeLa cells with the indicated genetic background dividing in contact with Wnt3a-beads. The bottom-right last four panels show wild-type HeLa cells dividing in contact with inactive Wnt3a-beads (iWnt3a, in which Wnt3a was inactivated by DTT treatment), or with EGF-coated beads.

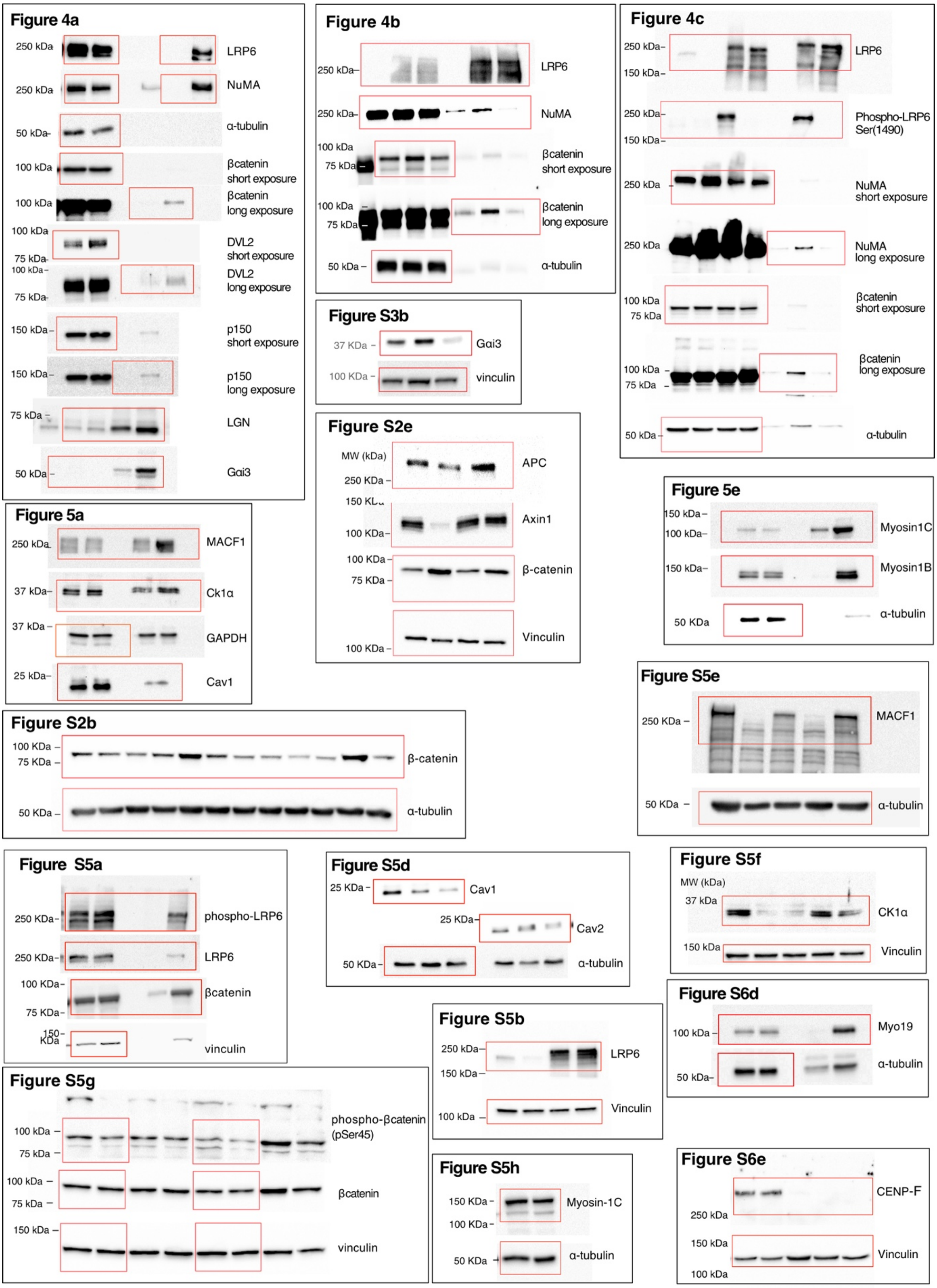

Supplementary Figure 9. Uncropped images of IB presented in the main and supplementary figures.

Supplementary Movie 1. TIRF Microscopy of myosin1C-YFP HeLa cells on Wnt3a-platform. Movie assembled with TIRF microscopy images of mitotic HeLa cells expressing an shCtrl and transfected with myosin1C-YFP landing on a Wnt3a-coated surface. Corresponding images are shown in Fig. 5g.

Supplementary Movie 2. TIRF Microscopy of myosin1C-YFP shLRP6 HeLa cells on Wnt3a-platform. Movie assembled with TIRF microscopy images of mitotic HeLa cells expressing an shLRP6 shRNA and transfected with myosin1C-YFP landing on a Wnt3a-coated surface. Corresponding images are shown in Fig. 5g.

Supplementary Table 1. List of selected protein enriched at Wnt3a-coated beads identified by proteomics

Gene name	Protein name	Log_2FC	$-Log_{10}$ (adjusted p-value)	Sequence coverage [%]
	Components of the Wnt transduction pathway			
CTNNB1	Catenin beta-1	Wnt3-ON	-	20.6
CSNK1G3	Casein kinase I isoform gamma-3	Wnt3-ON	-	26.2
CSNK1A1	Casein kinase I isoform alpha	2.78472	4,0247	30.0
CSNK2A1	Casein kinase II subunit alpha	1.49966	1.79454	13.7
CAV2	Caveolin-2	2.94776	1.5238	44.4
CAV1	Caveolin-1	2.68965	1.16921	61.8
RSPO3	R-spondin-3	2.6456	0.82835	19.5
SDC4	Syndecan-4	Wnt3-ON	-	37,9
SDC1	Syndecan-1	2.77773	1.82049	15.8
PPP2R1A	PPP2R1A-PPP2R2A-interacting phosphatase regulator 1	1.8809	1.43064	41.1
WNT3A	Protein Wnt-3a	1.38662	0.21918	25.6
	Mitotic proteins			
EPB41	Protein 4.1	Wnt3-ON	-	14.6
GNAI1	Guanine nucleotide-binding protein G(i) subunit alpha-1	1.84328	2.85755	42.7
GNAI2	**Guanine nucleotide-binding protein G(i) subunit alpha-2**	1.80061	1.66761	53.0
GNAI3	Guanine nucleotide-binding protein G(i) subunit alpha-3	1.94106	1.5259	46.9
NUMA1	Nuclear mitotic apparatus protein 1	1.88651	1.08253	54.5
KIF2A	Kinesin-like protein KIF2A	1.06542	0.81895	12.2
	Actin-related proteins			
MACF1	Microtubule-actin cross-linking factor 1	Wnt3-ON	-	6.5
ACTA1	**Actin, alpha skeletal muscle**	3.55266	3.04883	55.4
CAPZA1	F-actin-capping protein subunit alpha-1	1.64907	2.69225	76.6

MYO19	Unconventional myosin-XIX	Wnt3-ON	-	16.9
MYO1C	Unconventional myosin-Ic	1.48036	1.92433	45.0
MYO1B	Unconventional myosin-Ib	1.82029	1.52751	38.5

*Wnt3-ON when less than 5 ms/ms were identified in the control experiment. Log_2FC calculated with LFQ values when ms/ms>=5 in each condition.

Supplementary Table 2 List of shRNA sequences, RT-qPCR primers and cloning oligonucleotides

**Gene**	**Sh-RNA sequences**	**Vector**
Ctrl	AGACGAACAAGTCACCGACTT	pLKO.1-Puro
APC	CCCAGTTTGTTTCTCAAGAAA	pLKO.1-Puro
LRP6	GCAGATATCAGACGAATTTCT<br>GAACTATGATTCAGAACCTGT	pLKO.1-Puro
β-catenin	CGCATGGAAGAAATAGTTGAA<br>GCTTGGAATGAGACTGCTGAT	pLKO.1-Puro
Axin1	GGTGTTGGCATTAAAGGTG	pLKO.1-Puro
DVL1	GACGTGAACTTTGAGAACATG<br>CACGACCAAGGCCTATACAGT<br>ACGGAGTCCATGGTCAGTCAC	pLKO.1-Puro
DVL2	GCCACTGTGACTCTCACCAGCAGTGCCTG	pGFP-C-Lenti / GFP-Puromycin
DVL3	GATCTCGGATGACAATGCCAAGCTACCAT	pGFP-C-Lenti / Puromycin
Caveolin1	AGAGCTTCCTGATTGAGATTCAGTGCATC<br>TGGAAGGCCAGCTTCACCACCTTCACTGT	pLKO.1-Puro
Gαi1	TATGTATACTTGTATTAATAAA	pLKO.1-Puro
Gαi3	TACTGCTGATGTAGTATGATAA	pLKO.1-Puro
MACF1	GCCCACAACTAAAGGAATTAA<br>GCTGAGTTGATCTGGTTGAAT<br>CCCAAGCCACTATCCACTTTG<br>ACTTATGTGTCTTCGATTTAT	pLKO.1-Puro
Ck1-α	CATCTATTTGGCGATCAACAT<br>GCAAGCTCTATAAGATTCTTC<br>GAATTCATTGTCGGAGGGAAA<br>GCCTGCTTAATTGTGCTAGAA	pLKO.1-Puro
CENP-F	AAGAGAAGACCCCAAGTCATC<br>CAAAGACCGGTGTTACCAAG<br>CAGGAAAGACTAGCCCATATA<br>CAGAATCTTAGTAGTCAAGTA	pLKO.1-Puro
MIRO1	TAATCACATAGATAACGCCCCC	pLKO.1-Puro
MIRO2	GAGGTTGGGTTCCTGATTAAA	pLKO.1-Hygro
**Gene**	**RT-qPCR primer sequences**	**Source**
GAPDH-FW	CAACTACATGGTTTACATGTTC	MERCK
GAPDH-RV	GCCAGTGGACTCCACGAC	MERCK

Gai1	QT00023555	Qiagen
Gai3	QT00012509	Qiagen
TCF-FW	CTGACCTCTCTGGCTTCTACTC	MERCK
TCF-RV	CAGAACCTAGCATCAAGGATGGG	MERCK
RNF43-FW	GGTTACATCAGCATCGGACTTGC	MERCK
RNF43-RV	ATGCTGGCGAATGAGGTGGAGT	MERCK
DVL1-FW	CACCTCATCCAGACTCATCC	MERCK
DVL1-RV	TCAAAGTTCACGTCATTCACC	MERCK
MIRO1-FW	GACAAAGACAGCAGGCTGCCTT	MERCK
MIRO1-RV	TCGCTGAACACTCCACACAGGT	MERCK
MIRO2-FW	GCAGGTCAATGGACAGGAGAAG	MERCK
MIRO2-RV	TGTAGACGCTGGCACAATGTGC	MERCK
**Gene**	**Oligo sequences**	**Use**
LRP6-FW	CGCGCGTCGACATGGGGGCCGTCCTGAGGAGC	Cloning
LRP6-RV	GCGCGCGGATCCCCGGAGGAGTCTGTACAGGGAGA	Cloning
LRP6-ShRes	CTTTTGTTTTCACGGAGAGCAGACATAAGGCGTATTTCTCTGGAAACAA A	QuikChange
LRP6-M1	ATTTTGAACCCTCCACCAGCCCCAGCCACAGAGCGATCA	QuikChange
LRP6-M2	CGGCACTTTGCACCCCCCGCCGCACCCTGCAGCACAGATGTT	QuikChange
LRP6-M3	CCTGTGCCCCCACCTCCCGCACCCCGAAGCCAATAC	QuikChange
LRP6-M4	TATGAAAGCTGCCCACCTGCTCCATACACAGAGAGGAGC	QuikChange
LRP6-M5	CTCTACCCACCGCCACCCGCTCCCTGTACAGACTCCTCC	QuikChange

Supplementary Table 3 List of antibodies

Antibody name	Source	Dilution (Application)
**Afadin**	Mapelli Lab	1:200 (IF)
**APC**	Santacruz, sc-53165	1:500 (IB)
**Axin1**	Cell Signaling, C76H11	1:1000 (IB)
**α-tubulin**	Abcam, ab4074<br>Sigma, T6199	1:600 (IB)<br>1:300 (IF)
**β-catenin**	Millipore, 06-734	1:1000 (IB)
**β-catenin**	BD Transduction Laboratories, 610153)	1:500 (IF)
**Phospho-(β-catenin)**	Cell Signaling,9564	1:1000(IB)
**Caveolin-1**	Cell Signaling, D46G3	1:1000(IB)/1:500(IF)
**Caveolin-2**	BD Transduction Laboratories, 610684	1:250 (IB)
**CENP-F**	BD Transduction Laboratories, 610768	1:1000 (IB)

**Ck1α**	Cell Signaling, BK2655S	1:1000 (IB)
**Dischevelled2**	Cell Signaling, 3224S	1:1000 (IB)
**Dischevelled3**	Cell Signaling, 3218S	1:1000 (IB)
**GAPDH**	Santa Cruz Biotechnology, sc-32233	1:1000 (IB)
**Gαi1**	Santa Cruz Biotechnology, sc-56536	1:100 (IF)
**Gαi3**	BD Transduction Laboratories, 610201	1:10.000 (IB)
**γ-tubulin Cy3**	Sigma-Aldrich, C7604	1:200 (IF)
**γ-tubulin**	Abcam, ab11316	1:100 (IF)
**LGN**	Mapelli Lab	1:500 (IB/IF)
**LRP6**	Cell Signaling, 3395S	1:1000 (IB)
**Phospho-Ser1490-Lrp6**	Cell-signaling, 2568S	1:1000 (IB)
**L1CAM (UJ127.11)**	Santa Cruz Biotechnology, sc-53386	1:20 (IF)
**MACF1**	Invitrogen, PA5-66976	1:750 (IB)
**myosin1B**	From Evelyne Coudrier Lab	1:1000 (IB)
**myosin1C**	Santacruz, sc-136544	1:1000 (IB)
**myosin-19**	Abcam, ab174286	1:2000 (IB)
**NuMA**	Mapelli Lab	1:200 (IB) / 1:3000 (IF)
**p150Glue**	BD Transduction Laboratories, 610473	1:500 (IB) / 1:100 (IF)
**TOM20**	Santa Cruz Biotechnology, sc-17764	1:500 (IF)
**Vinculin**	Cell Signaling Technology,13901	1:10000 (IB)
