# Supplementary material for: Localized Wnt-signaling promotes asymmetric NuMA-dependent oriented divisions and unequal apportioning of mitochondria
Source: Nat Commun. 2025 Nov 27;16:10690. doi: 10.1038/s41467-025-65775-z (PMC12660846; doi:10.1038/s41467-025-65775-z)
Supplement: Supplementary file 2 — Description of Additional Supplementary Information [file 41467_2025_65775_MOESM2_ESM.pdf]

Description of Additional Supplementary Files

File Name: Supplementary Movie 1
Description: **TIRF Microscopy of myosin1C-YFP HeLa cells on Wnt3a-platform**. Movie assembled with TIRF microscopy images of mitotic HeLa cells expressing an shCtrl and transfected with myosin1C-YFP landing on a Wnt3a-coated surface. Corresponding images are shown in Fig. 5g.

File Name: Supplementary Movie 2
Description: **TIRF Microscopy of myosin1C-YFP shLRP6 HeLa cells on Wnt3a-platform.** Movie assembled with TIRF microscopy images of mitotic HeLa cells expressing an shLRP6 shRNA and transfected with myosin1C-YFP landing on a Wnt3a-coated surface. Corresponding images are shown in Fig. 5g.
